# Lithium Protects Against Anaesthesia Neurotoxicity In The Infant Primate Brain

**DOI:** 10.1038/srep22427

**Published:** 2016-03-08

**Authors:** Kevin K. Noguchi, Stephen A. Johnson, Lauren E. Kristich, Lauren D. Martin, Gregory A. Dissen, Emily A. Olsen, John W. Olney, Ansgar M. Brambrink

**Affiliations:** 1 Department of Psychiatry, Washington University School of Medicine, St. Louis, MO, US; 2 Division of Comparative Medicine, Oregon National Primate Research Center, Oregon Health & Science University, Beaverton, Oregon, US; 3 Division of Neuroscience, Oregon National Primate Research Center, Oregon Health & Science University, Beaverton, Oregon, US; 4 Department of Anesthesiology & Perioperative Medicine, Oregon Health & Science University, Portland, Oregon, US

## Abstract

Exposure of infant animals, including non-human primates (NHPs), to anaesthetic drugs causes apoptotic death of neurons and oligodendrocytes (oligos) and results in long-term neurodevelopmental impairment (NDI). Moreover, retrospective clinical studies document an association between anaesthesia exposure of human infants and significant increase in NDI. These findings pose a potentially serious dilemma because millions of human infants are exposed to anaesthetic drugs every year as part of routine medical care. Lithium (Li) at clinically established doses is neuroprotective in various cerebral injury models. We therefore investigated whether Li also protects against anaesthesia neurotoxicity in infant NHPs. On postnatal day 6 NHPs were anaesthetized with the widely used anaesthetic isoflurane (ISO) for 5 h employing the same standards as in a human pediatric surgery setting. Co-administration of Li completely prevented the acute ISO-induced neuroapoptosis and significantly reduced ISO-induced apoptosis of oligodendroglia. Our findings are highly encouraging as they suggest that a relatively simple pharmacological manipulation might protect the developing primate brain against the neurotoxic action of anaesthetic drugs while not interfering with the beneficial actions of these drugs. Further research is needed to determine Li’s potential to prevent long-term NDI resulting from ISO anaesthesia, and to establish its safety in human infants.

Anaesthetic drugs are essential for optimal patient care in pediatric and obstetric medicine, but they are also exogenous neuroactive drugs that have the potential to disrupt brain circuitry and adversely affect the functional attributes of the developing brain. A growing body of evidence documents that several classes of drugs, including anaesthetic, but also alcohol and anti-epileptic drugs, trigger apoptotic cell death in the developing brains of several animal species, including non-human primates (NHPs)^1^,^2^,^3^,^4^,^5^,^6^. Adverse long-term neurobehavioral consequences of anaesthesia exposure in infancy has also been described in both rodents reviewed in^7^ and NHPs^8^. The cell death response in fetal or infant macaques exposed to alcohol or anaesthetic drugs manifests as widespread death of two cell types - neurons and oligodendrocytes (oligos)^6^,^9^,^10^,^11^,^12^,^13^. Oligos are responsible for generating and maintaining the myelin sheath, which is essential for normal neuronal function. Developmental loss of neurons, compounded by simultaneous loss of oligos, is a type of brain injury that potentially could contribute to long-term neurobehavioral impairment. Specifically, in infant NHPs following isoflurane anaesthesia (ISO; 5 h; surgical tolerance) we observed widespread apoptotic cell death throughout both, the gray and white matter^1^,^9^. Compared to brains of infant NHPs that never received ISO (controls), brains after a 5-hour ISO anaesthesia showed a >10-fold increase in neuronal apoptosis^1^, and a loss of about 6% of their total oligo cell population to apoptotic cell death^9^. Concern over these animal findings was recently heightened by a series of studies^14^,^15^,^16^,^17^,^18^,^19^,^20^ documenting that exposure of human infants prior to three years of age to brief anaesthesia is associated with a significant increase in risk for long-term learning disabilities.

These findings pose a potentially serious problem because: 1) Millions of human fetuses and infants, including premature infants, are exposed to anaesthetic drugs every year; 2) Anaesthetic drugs are essential for the delivery of optimal medical care; 3) All anaesthetic drugs currently in use have been shown to trigger neuroapoptosis in the developing mammalian brain.

A potential solution to this problem would be to develop a neuroprotective drug that: 1) Is safe; 2) Does not interfere with the beneficial actions of anaesthetic drugs; 3) Can prevent anaesthetic drugs from triggering neuro- and oligo-apoptosis.

There is evidence in infant mice that an intracellular kinase signaling system (extracellular signal-regulated protein kinase - ERK) that is known to play an important role in cell survival is adversely affected by alcohol and anaesthetic drugs. These drugs rapidly block activation (phosphorylation) of ERK and this occurs immediately before immunohistochemical evidence for apoptosis appears^21^,^22^. We have shown that lithium (Li) promotes phosphorylation of ERK, and prevents alcohol^21^ or anaesthetic drugs^22^ from suppressing this phosphorylation process, and also prevents these drugs from triggering neuroapoptosis in the infant mouse brain. The present study was undertaken to determine whether Li exerts a similar neuroprotective action against anaesthesia neurotoxicity in the infant primate brain.

## Results

To evaluate the neuroprotective properties of Li in the developing primate brain, we exposed infant macaques to ISO (n = 5), ISO + Li (n = 5) or no anaesthesia (Control; n = 5). Animals were anaesthetized for 5 hours and then recovered and observed for 3 hours. Control animals received identical handling as those randomized to undergo general anaesthesia but were then returned to their dams. At 8 hours after time zero all animals were deeply anaesthetized and perfusion-fixed to prepare the brains for systematic analysis. On the day of the experiment, animals were comparable regarding age (5.8 ± 0.4, 6.6 ± 0.9 and 5.6 ± 1.1 days [mean ± SD], respectively [range 5–7 days]) and body weight (550 ± 27, 504 ± 54 and 511 ± 48 g [mean ± SD], respectively [range 433–584 g]). ISO anaesthesia (average end-tidal concentration = 1.8%; range 1.5–3.0%) was well tolerated by all infant animals and physiologic parameters remained within the normal species-specific range throughout the five-hour exposure period. Animals that received Li co-treatment required the same ISO doses to achieve the targeted depth of anaesthesia (surgical tolerance) as those that were not co-treated with Li. Similarly, anaesthetized animals with and without Li co-treatment were weaned from the ventilator within minutes and the trachea was extubated within 15 min after discontinuation of ISO. Animals in both groups were equally responsive and able to drink formula milk when offered. Animals that had received Li together with ISO appeared slightly less active during the recovery period compared to those that had received ISO only. Table 1 summarizes the Li serum levels obtained from the animals that were co-treated with Li during the ISO anaesthesia. In all but one animal the target Li levels were achieved with the predetermined dose regimen and the serum levels remained within a narrow range throughout the 8 h experiment (0.9 to 1.4 mEq/L). One animal had higher Li serum levels (range 1.7 to 2.2 mEq/L) despite receiving the same dosage regimen. The Li serum levels in this animal were consistently in a range that can be associated with toxic side effects in a long-term psychiatric treatment context. However, neither this animal, nor any of the other Li treated animals showed clinical signs of toxicity (such as arrhythmia, polyuria, ataxia, tremor, nausea, diarrhea) either prior to induction of, or during the 3-hour observation period after the end of ISO anaesthesia. Nevertheless, the 3-hour post-anaesthesia observation time was rather short and would not allow us to exclude any delayed Li-induced effects. No complications occurred during euthanasia and *in-vivo* tissue fixation.

**Figure 1:**
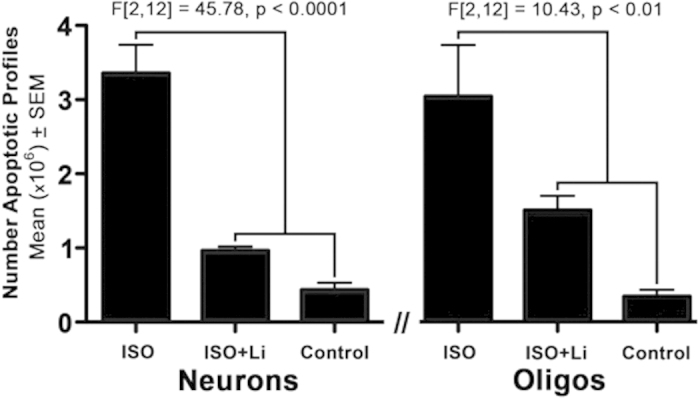
Number of apoptotic neurons or oligos under each treatment condition (n = 5 per group). Data were evaluated by two one-way ANOVAs which revealed a statistically significant difference between groups for both neurons and oligos. Posthoc analysis revealed the mean (±SEM) number of apoptotic neurons per brain in the isoflurane (ISO) group was significantly greater than for the ISO + lithium (Li) or the control group (P < 0.001 for both). The mean number of apoptotic neurons for the ISO + Li group was not significantly different from controls. The number of apoptotic oligos for the ISO group was significantly different from the ISO + Li or control group (P < 0.05 and 0.01 respectively) and the number of apoptotic oligos for the ISO + Li group versus the control group was not significantly different.

Quantitative evaluation of brain sections by unbiased stereological methods revealed that the group exposed to ISO had a large increase in the mean (±SEM) number of apoptotic neurons (3.36 × 10^6^ ± 0.38 × 10^6^) compared to the group that received no anaesthesia (controls; 0.44 × 10^6^ ± 0.11 × 10^6^) (P < 0.001). In contrast, the group that received Li co-treatment during ISO had a much lower mean number of apoptotic neurons (ISO + Li; 0.96 × 10^6^ ± 0.05 × 10^6^) compared to ISO alone (P < 0.001). In fact, the mean numbers of apoptotic neurons in ISO + Li were not significantly different from controls (Fig. 1). ISO also caused a large increase in the mean (±SEM) number of apoptotic oligos (3.04 × 10^6^ ± 0.69 × 10^6^) compared to controls (0.34 × 10^6^ ± 0.07 × 10^6^) (P < 0.01) (Fig. 1). The mean for the ISO + Li group (1.5 × 10^6^ ± 0.19 × 10^6^) was significantly reduced compared to the ISO group (P < 0.05). While the number of apoptotic oligos was not significantly different from the control value, the degree of protection for oligos was less than that for neurons. Table 2 summarizes the apoptosis counts for each individual animal in the Li co-treated group. A Pearson’s r revealed no linear correlation between Li serum levels (Table 1) and the number of apoptotic cells (Table 2) (p > 0.05).

**Figure 2:**
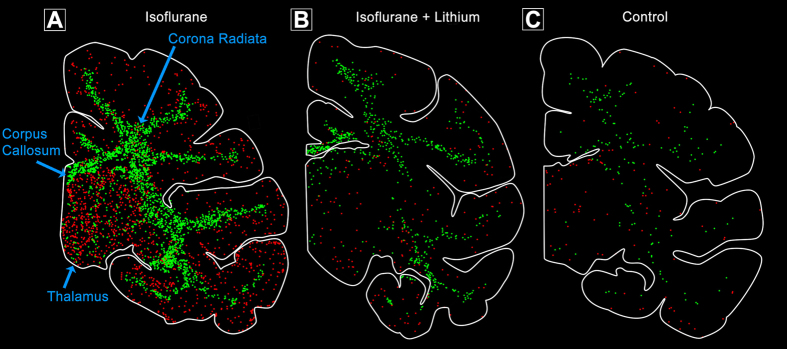
Pattern of neuronal and glial apoptosis following isoflurane anaesthesia in infant monkey at the level of the thalamus. Computer generated duel plots show apoptotic (AC3-positive) neurons (red) and glia (green) in animals after a 5-hour isoflurane anaesthesia (**A**) 5 hours of isoflurane plus lithium co-treatment (**B**) or no anaesthesia (control; (**C**)). Isoflurane produces high levels of neuronal apoptosis in the thalamus and cortex whereas oligo apoptosis is concentrated in the white matter including the corpus callosum and corona radiata. Lithium co-treatment during isoflurane anaesthesia dramatically reduces apoptosis in both cell types similar to that seen in controls (no anaesthesia).

**Figure 3:**
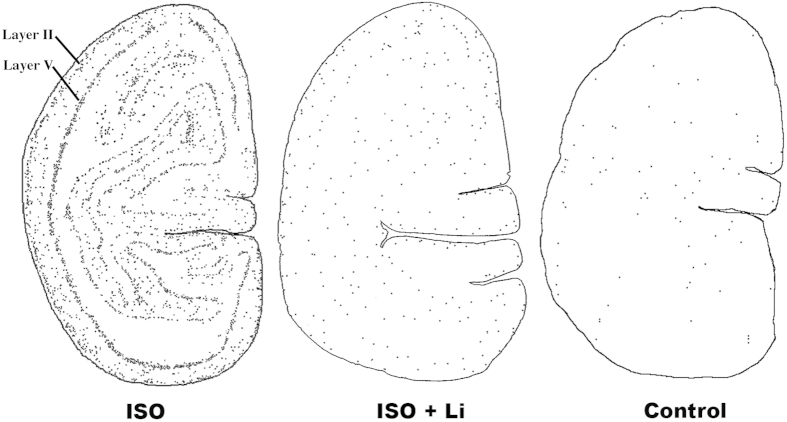
Pattern of neuronal apoptosis induced by ISO anaesthesia at the level of the primary visual cortex. Computer generated plots depict the density and location of apoptotic neurons (black dots) following each of the three treatment conditions, ISO (isoflurane), ISO + Li (isoflurane + lithium) or control. The brain exposed to ISO displays a very high density of apoptotic neurons, and they are distributed in an organized linear pattern corresponding to the location of layer II and V neurons. In contrast, the ISO + Li and the control brains have a much lower density of apoptotic neurons and the linear pattern is only faintly recognizable. In the temporal and somatosensory cortices ISO also caused an organized linear pattern of neuronal degeneration which was much less evident in the ISO + Li and control brains.

**Figure 4:**
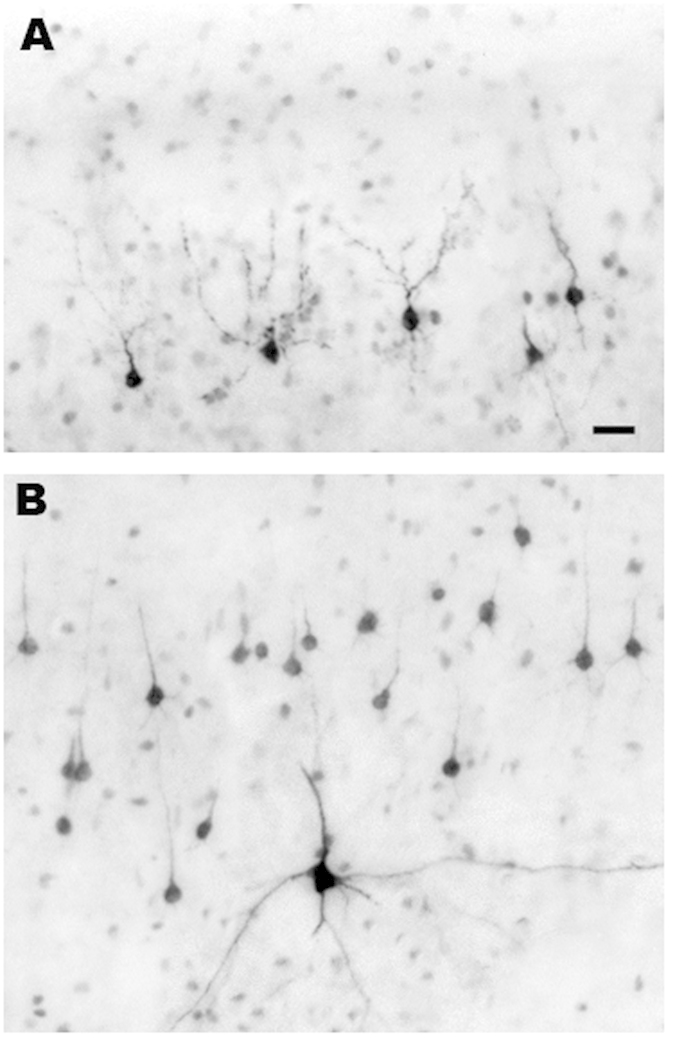
Histological sections from the primary visual cortex of an infant monkey exposed to ISO, illustrating the appearance of neuronal profiles that are stained by activated caspase 3 (AC3), a selective marker for apoptosis. As shown in Fig. 2, the neurotoxic action of ISO selectively impinges on neurons in layers II and V of the primary visual cortex. The dark profiles in panel (**A**) are AC3-positive neurons undergoing apoptotic cell death in layer II, and those in panel (**B**) are AC3-positive neurons undergoing apoptotic cell death in layer V. The dying neurons in layer II panel (**A**) are GABAergic inhibitory neurons. Most of those in layer V panel (**B**) are small pyramidal neurons that are thought to be glutamatergic excitatory neurons. The single larger multipolar profile in panel (**B**) has the morphological characteristics of a Martinotti neuron^35^, which is believed to exert an inhibitory action. Bar in A = 20μm for both panels (**A,B**).

**Figure 5:**
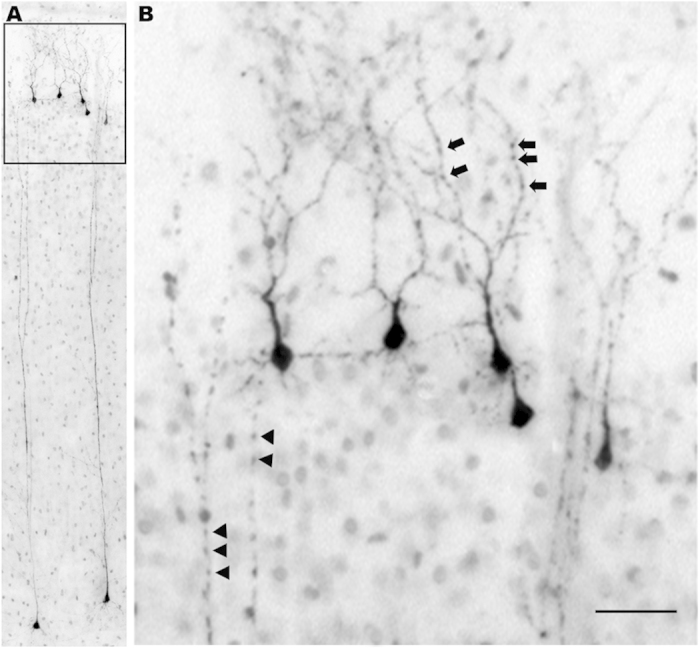
Neuronal degeneration in the temporal cortex following isoflurane anaesthesia. Panel (**A**) shows the typical pattern of apoptotic cell death which features a row of dying neurons in layer II superficial to the cortical surface, and a second row of large pyramidal neurons in deep cortical layers. The layer II neurons have a bifurcated trunk that feeds branches into layer I all the way up to the cortical surface, and are thought to be GABAergic inhibitory neurons. The cell bodies of the vulnerable deep-layer pyramidal neurons are typically located in either layers IV, V or VI, and send very long apical dendrites all the way up to layers II and I. As these neurons, both superficial and deep, undergo apoptosis, an early sign of degeneration is beading of the terminal dendritic processes. Segmental beading is the first step and the next step is disintegration of the dendritic tree into many small fragments that litter the neuropil. In panel (**B**) (magnified view of boxed region from panel (**A**) segmental beading is seen in the terminal dendrites of both the deep pyramidal neurons (arrow heads) and the Layer II superficial neurons (arrows). Bar in B = 30 μm in panel (**B**) and 150 μm in panel (**A**).

**Figure 6:**
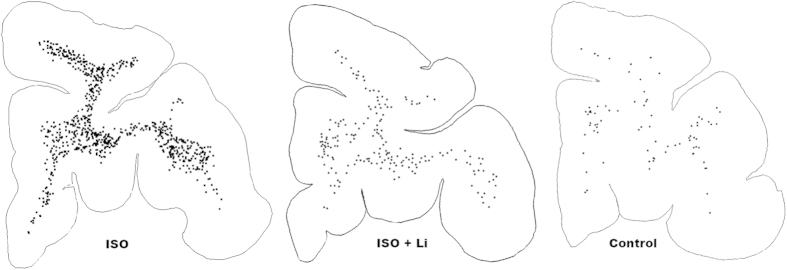
Pattern of oligo-apoptosis induced by isoflurane anaesthesia in the infant monkey brain. Here we show computer plots at the level of the prefrontal cortex depicting the density and location of apoptotic oligos (black dots) under each treatment condition. The apoptotic oligo profiles are confined to a white matter distribution under each treatment condition, but are present in high density in the ISO-exposed brain, compared to lower and lowest density in the ISO + Li and control brains, respectively. The distribution of degenerating oligos shown here is representative of the distribution throughout the brain; they are diffusely and relatively evenly distributed throughout all white matter pathways.

**Figure 7:**
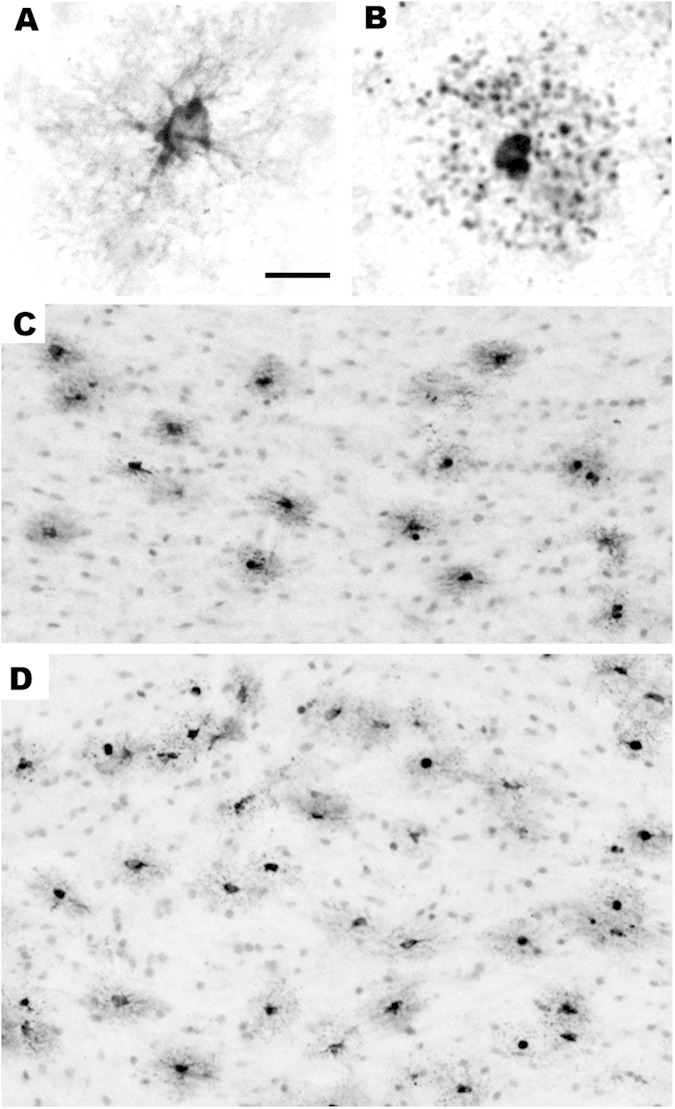
Histological sections illustrating the appearance of degenerating oligos stained by AC3 or myelin basic protein (MBP). We have shown^9^,^11^ that the white matter cells that undergo apoptosis when exposed to isoflurane co-label for AC3 (apoptosis marker) and MBP. This identifies these cells as oligodendrocytes (oligos) because oligos are the only cell type in the brain that contains MBP. Panels (**A,B**) are both stained with antibodies to MBP. A illustrates the appearance of a normal healthy oligo for comparison with the cell in B that is in an advanced stage of apoptosis following exposure to isoflurane. As the oligo degenerates, its processes become fragmented into many small dark balls that distribute around the cell body in a star burst configuration (**B**). When oligos undergo apoptosis they stain selectively and intensely with antibodies to AC3. Therefore, this is the method we employ for mapping and quantifying apoptotic oligos under various treatment conditions. Panels (**C,D**) illustrate the appearance of apoptotic oligos following isoflurane exposure in two major white matter tracts, the corpus callosum (**C**) and internal capsule (**D**). In each of these white matter pathways apoptotic oligos stand out as dark profiles which are typically surrounded by a smudgy circle of tissue discoloration which reflects leakage of AC3 into the neuropil from their disintegrating processes. Bar in A = 15 μm in A and B, and = 75 μm in C and 100 μm in (**D**).

To confirm the identity of brain cells undergoing apoptosis and to clarify whether some cell populations are more vulnerable than others we applied a battery of histological staining procedures, including IHC staining with reagents specific for various CNS cell types or cell death processes. Antibodies to AC3 detected all cells dying by apoptosis in either gray matter or white matter regions of the brain. DeOlmos cupric silver stain detected cells that were dying or dead, and it marked the same populations of cells that stained positive for AC3. The vast majority of cells in gray matter regions were considered to be neurons based on morphological features but also because they co-labeled for AC3 and NeuN, a specific neuronal marker. ISO anaesthesia induced widespread neuronal apoptosis affecting multiple subcortical nuclei including the thalamus and basal ganglia, as well as the cerebral cortex. In contrast, lithium co-treatment dramatically reduced ISO-induced neuronal apoptosis across all brain regions, and the the density (counts / mm^3^) and distribution of AC3-positive cells in brains of lithium-treated infants was similar to that seen in controls (Figs 2 and 3). ISO-induced neuronal cell death in several cortical regions showed an organized, linear pattern which corresponded to the location of layer II and V neurons (Fig. 3). Morphologic analysis of somatosensory, temporal and primary visual cortices revealed that programmed cell death predominantly affected neuronal populations that are engaged in sensory information processing (Figs 4 and 5). In visual cortex (Fig. 4) the majority of dying neurons in layer II were GABAergic inhibitory neurons (Fig. 4, Panel A), while those in layer V typically were small pyramidal glutamatergic excitatory neurons (Fig. 4, Panel B). Similarly, in temporal cortex dying neurons were apparent in layer II which represent GABAergic inhibitory neurons that send branches into layer I at the cortical surface (Fig. 5, Panel B), while in deeper layers (IV–VI) ISO induced apoptosis affected predominantly pyramidal neurons that send long apical dendrites into layers II and I (Fig. 5, Panel A). As an early sign of degeneration many AC3-positive neurons showed ‘beading’ of the terminal dendritic processes (Fig. 5, Panel B). Somatosensory cortex showed identical patterns (data not shown). Apoptotic oligo profiles were distributed diffusely and in a relatively even pattern throughout all white matter regions including the corpus callosum and corona radiata (Figs 2 and 6). Apoptotic profiles in the white matter were determined to be oligos because they co-labeled for AC3 and myelin basic protein, a molecule that is not contained in any CNS cell other than oligos (Fig. 7). In addition, these apoptotic profiles did not stain positive for antibodies specific for astrocytes or microglia (data not shown).

## Discussion

Our findings demonstrate that intravenous administration of Li to infant rhesus macaques, at a well-tolerated dose, while they are being exposed to a surgical plane of ISO anaesthesia for 5 h, almost completely prevents the acute neuroapoptosis response that predictably occurs in the absence of Li co-exposure. In addition, apoptotic death of oligos, which also predictably occurs following ISO anaesthesia, was significantly, but not as dramatically, reduced by Li co-exposure. Unequal protection against neuroapoptosis and oligoapoptosis probably signifies that the underlying mechanisms, while very similar, are not identical in all respects. It will be important to clarify differences in the underlying mechanisms, and develop a more complete understanding of all mechanisms involved, as this may lead to the development of neuroprotective agents that can totally prevent both toxic reactions.

These findings are potentially promising in that the dose of Li used is one that maintains blood levels of Li in a range (0.6 to 1.2 mEq/L) considered safe and therapeutic for human patients undergoing long-term Li therapy for psychiatric conditions. If a single exposure to Li at a seemingly safe dose can eliminate or substantially reduce the neurotoxic and oligotoxic response to 5 hours of isoflurane anaesthesia in a highly controlled and clinically relevant infant NHP model, a similar protective effect may be expected in human infants that are exposed to general anaesthesia. While only a minority of human infants require 5-hour long anaesthesia, testing the effects of Li co-treatment during experimental anaesthesia of such duration allowed us to determine that Li co-treatment protects for at least 5 hours against ISO-induced apoptosis in NHP infants. If similar effects could be reproduced in human infants, potentially detected by a non-invasive biomarker, Li co-treatment may become a protective strategy that potentially could become part of the anaesthetic plan for the majority of infants who undergo shorter, as well as longer anaesthesia (0–5 hours) in order to tolerate surgical or diagnostic procedures.

The mechanism(s) underlying Li’s protective action against apoptotic death of brain cells are not fully understood, but there is strong evidence that various drugs that have apoptogenic activity in the developing brain (alcohol, anaesthetic and anti-epileptic drugs) acutely block phosphorylation of ERK^3^,^21^,^22^,^23^,^24^, an intracellular kinase signaling system that is known to promote survival of brain cells during critical stages of development. Developing neurons are programmed to commit suicide (undergo apoptosis) if support mechanisms fail and prevent them from meeting developmental milestones. Thus, blockade of ERK phosphorylation by these drugs may be an event that can derail developing brain cells from their normal survival pathway, thereby ensuring an opposite fate - cell death by apoptosis. We have demonstrated in the *in vivo* infant mouse brain that Li promotes ERK phosphorylation and prevents alcohol and anaesthetic drugs from suppressing this process, and also prevents these drugs from triggering neuroapoptosis^21^,^22^. Regulation of cell survival during development is accomplished by a complex sequence of intracellular signaling mechanisms that are operative both upstream and downstream of ERK. Alcohol and anaesthesia-induced apoptosis are known to be bax-dependent^25^ and to be triggered by translocation of bax protein to mitochondria where the apoptosis cascade is unleashed^26^. In contrast to other protective drugs identified thus far including melatonin^27^, L-Carnitine^28^ and agents that suppress oxidative free radical formation^29^ that are thought to interact at points downstream of bax translocation (an apoptotic trigger), the ERK system appears to play an important role in events that precede unleashing of the apoptotic cascade. Therefore, this signaling system can be viewed as an important intracellular target for new drug development aimed at providing safe and effective protection against developmental anaesthetic neurotoxicity.

How promising Li itself may be as a candidate for protecting the developing brain against anaesthesia neurotoxicity will depend on whether future research can establish that it is superior to other protective strategies in both efficacy and safety. Li has been used for decades in the treatment of psychiatric conditions, primarily bipolar disorder. It has also been used in the treatment of childhood psychiatric conditions, but there has never been any medical indication for its use in infants. Human fetuses are sometimes exposed chronically to Li during a pregnancy in which the mother requires daily Li therapy for bipolar disorder. There is evidence^30^ that fetal exposure to Li in the first trimester is associated with a slightly increased risk for cardiac anomalies, but we are not aware of any reports of deleterious effects due to Li exposure in later stages of gestation. We have observed in infant mice that a single dose of Li suppresses the rate of neuroapoptosis that occurs naturally in the developing brain^21^,^22^. Therefore, it is possible that chronic exposure of the developing brain to Li might interfere with the natural process by which the brain rids itself of redundant or dysfunctional elements. However, if Li exposure on a brief one-time basis can protect the primate brain against anaesthesia-induced loss of millions of developing neurons and oligos, this would be a substantial benefit compared to the potentially minimal risk associated with very brief interference in the natural debridement process.

An issue requiring further study is whether Li provides permanent protection against apoptotic cell death or merely delays the onset of cell death, e.g. by changing kinetics or time course of the apoptotic cascade, so that apoptosis after ISO + Li does not happen within the time interval examined in our study, but rather much later. This issue was recently addressed by Sadrian *et al*.^31^, who confirmed the prior finding^21^,^32^ that Li prevents alcohol from triggering acute neuroapoptosis in the infant mouse brain. These authors went further and demonstrated that Li also prevents long-term electrophysiological and neurobehavioral disturbances that alcohol causes in the absence of Li co-treatment. Similarly, we have shown that Li provides both, immediate and long-term protection against neural progenitor cell apoptosis induced by dexamethasone in infant mouse cerebellum^33^.

It will be important in future research to determine in NHP subjects whether Li not only protects against the acute brain injury induced by anaesthetic drugs, but also can prevent long-term neurobehavioral disturbances that have been shown to occur^8^ following anaesthesia exposure of the infant primate brain. Such research also would require carefully designed control groups that, among other issues, allow determination of potential long-term effects of neuronal plasticity that may compensate for initial deleterious effects of anaesthesia exposure independent of any Li co-treatment.

There are critical stages during development when the brain is exquisitely sensitive to the influence of neuroactive drugs, because these drugs interact with brain cells while they are undergoing plasticity changes required for integration into functional neural networks. Exogenous drugs like many of today’s anaesthetics are an unphysiological influence that can permanently alter the outcome of this integration process, especially if their influence is so extreme that it causes brain cells to die by apoptosis and be deleted from the circuitry that they were biologically intended to support. The Li findings described herein suggest that it may be possible by a relatively simple pharmacological manipulation to prevent anaesthetic drugs from adversely altering the integration process while not interfering with the beneficial actions of these drugs. It must be remembered, however, that Li itself is a neuroactive drug that has the potential to interact in poorly understood ways with developing brain circuits, so it would not be prudent to introduce Li as a protective therapy for human infants, including premature infants, until additional research has adequately proven its safety for this purpose.

## Materials and Methods

All animal protocols received approval by the institutional animal care and use committee of Oregon National Primate Research Center (Beaverton, OR) and were conducted in full accordance with the Public Health Service Policy on Humane Care and Use of Laboratory Animals. All procedures were performed according to the same methods and standards that are employed in a human pediatric surgical setting.

### General anaesthesia

Six day-old infant rhesus macaques (n = 5/group) received either ISO anaesthesia for 5 hours, ISO together with IV Li (ISO + Li), or no drug (Control). The control group and group exposed to ISO alone are animals that served as subjects in a prior study (6) in which it was demonstrated that ISO exposure results in a marked increase in the rate of apoptotic cell death compared to the natural rate in drug naive controls. Re-using NHP subjects from this prior study is justified in that once the neurotoxicity of ISO has been demonstrated in rodent studies and confirmed in an NHP study, using the requisite number of animals to provide statistically significant results, it is not ethically defensible to sacrifice additional NHP infants to re-address the same scientific question (does ISO trigger neuroapoptosis in the NHP infant brain). ISO anaesthesia was administered as previously described^6^,^9^. Briefly, ISO was administered at a concentration (average 1.8%, range 1.5–3.0%) tightly regulated to maintain a surgical plane of anaesthesia (no movement and not more than 10% increase in heart rate or blood pressure in response to a profound mosquito-clamp pinch at hand and foot; checked every 30 min). During general anaesthesia (ISO or ISO + Li), animals were mechanically ventilated via an endotracheal tube and their physiologic status was extensively monitored and maintained using the same techniques as in previous experiments (including hemodynamics, ventilation, blood gases, metabolic status, temperature^6^,^9^). After anaesthetic exposure, animals were kept for 3 h in an infant monkey incubator, were visually monitored and were fed formula as tolerated. At the end of the 3-h observation period the animals were deeply anaesthetized using high-dose pentobarbital and immediately transcardially perfusion-fixed to prepare the brain for histopathologic analysis. Animals in the control group (no drug) underwent a similar procedure including an IV cannula, physiologic measurements and a period of handling to simulate the environment that the other animals experienced before induction of anaesthesia. Control animals were then returned to their dams until final measurements and transcardial perfusion under deep anaesthesia 8 h after time zero.

### Lithium administration

Li Carbonate (pharmaceutical grade) was compounded at 0.15 mEq/ml (University Compounders LLC, Portland OR) for intravenous application. Animals randomized to the ISO + Li group were pretreated with Li Carbonate at 75 min (0.75 mEq/kg, IV), and at 10 min (0.25 mEq/kg, IV) prior to the start of ISO. During anaesthesia, animals received additional Li Carbonate (0.15 mEq/kg, IV) at 1.5 h and 4.5 h after induction, respectively. The doses were determined based on results from pilot experiments and aimed to rapidly achieve and maintain Li blood levels in a range that is considered non toxic in a psychiatric treatment context. In establishing the desired dose range we took into consideration that Li is administered to psychiatric patients chronically on a daily basis and Li blood levels are typically monitored 12 h after the most recent dose. When treating acute mania, the targeted range for Li blood levels is 1.0 to 1.5 mEq/L; for maintenance therapy after the acute condition is brought under control, the targeted range for Li blood levels is 0.6 to 1.2 mEq/L. In either the acute or maintenance treatment context in psychiatry, the dose of Li administered at time zero must be one calculated to yield blood Li levels 12 h later that are in a certain range, so it is understood that the blood levels will be in a higher range in the interim period from time zero to time 12 h. In the context of using Li for neuroprotection against developmental anaesthesia toxicity, the goal is to maintain Li blood levels for a relatively brief period while the anaesthetic drug is in contact with brain cells. Thus, blood levels that are considered safe for humans in the interval from time zero to time 12 h would be an appropriate goal for Li neuroprotection against anaesthesia toxicity. Therefore, we conducted pilot experiments and based on the results we chose a Li dosing schedule (see above) calculated to maintain Li blood levels in the range from 1.0 to 1.4 mEq/L during the 5-h period of anaesthesia exposure. Blood samples to determine Li serum levels were obtained before Li administration (baseline) and at 2, 4, 6 and 8 h after induction of anaesthesia.

### Histopathologic analysis

At the end of the 3-h observation period (8 h from time zero) all subjects were euthanized by methods as described previously in detail^6^,^9^. The perfusion-fixed brains were then removed and studied for evidence of apoptotic death of brain cells. A battery of histological procedures, previously described in detail^6^,^9^,^10^,^11^,^12^,^13^,^34^, was applied to characterize the cell death process, identify the cell types affected and evaluate the pattern of injury. For quantitative assessment, we cut coronal serial sections (vibratome) across the entire rostro-caudal extent of the brain. From these sections, in an unbiased manner, we selected sections at 2 mm intervals (approximately 30 sections per brain) for antigen retrieval and IHC staining with antibodies to activated caspase 3 (AC3), a well-established marker for detection of brain cells undergoing apoptosis^6^,^9^,^10^,^11^,^12^,^13^,^34^. An experienced neurohistologist who was blinded to the experimental conditions counted all cellular profiles that stained positive for AC3, using a computer-assisted Microbrightfield Stereo-Investigator system to record the location and number of dying cells, and the quantitative dimensions of the counting field. Cell-type specific quantitative assessments for apoptotic neurons and apoptotic oligos was conducted as illustrated previously^6^,^9^,^10^,^11^,^12^,^13^,^34^ based on AC3 stain displaying the full cell body and processes of both cell types, and the distinctly different morphologic features for the two cell populations (see Figs 3, 4 and 6 for representative examples). Moreover, the dying oligos are confined almost exclusively to white matter and the dying neurons are confined exclusively to gray matter.

### Statistical evaluation

Data are presented as mean ± standard error of the mean (SEM). The data were analyzed by one-way ANOVA and posthocs using the Newman-Keuls multiple comparison method, with the aid of GraphPad Prism software, version 4.0a (GraphPad Software, Inc., La Jolla, CA).

## Additional Information

**How to cite this article**: Noguchi, K. K. *et al*. Lithium Protects Against Anaesthesia Neurotoxicity In The Infant Primate Brain. *Sci. Rep.* **6**, 22427; doi: 10.1038/srep22427 (2016).

## Figures and Tables

**Table 1:** Lithium serum levels (meq/L).

Animal No.	Experimental Time (hours)					
	zero	2.0	4.0	6.0	8.0	Median (Range)
*Iso* + *Li #1*	0	0.9	1.2	1.1	0.9	**1.00 (0.9–1.2)**
*Iso* + *Li #2*	0	0.9	1.4	1.3	1.2	**1.25 (0.9–1.4)**
*Iso* + *Li #3*	0	1	1.2	1.1	0.9	**1.05 (0.9–1.2)**
*Iso* + *Li #4*	0	1.9	2.2	2	1.7	**1.95 (1.7–2.2)**
*Iso* + *Li #5*	0	1.2	1.3	1.3	1	**1.25 (1.0–1.3)**

**Table 2:** Density (counts / mm^3^) of apoptotic cells per brain.

Animal No.	AC3 + Density/Brain		
	Neurons	Oligos	Neurons + Oligos
*Iso* + *Li #1*	16.82	22.57	39.39
*Iso* + *Li #2*	18.47	36.14	54.61
*Iso* + *Li #3*	16.5	31.07	47.57
*Iso* + *Li #4*	18.91	17.86	36.77
*Iso* + *Li #5*	17.88	30.24	48.12
